# Transgenerational plasticity of exploratory behavior and a hidden cost of mismatched risk environments between parental sexes

**DOI:** 10.1038/s41598-023-46269-8

**Published:** 2023-11-13

**Authors:** Denis Meuthen, Arash Salahinejad, Douglas P. Chivers, Maud C. O. Ferrari

**Affiliations:** 1 Department of Biology, University of Saskatchewan, 112 Science Place, Saskatoon, SK S7N 5E2 Canada; 2 Evolutionary Biology, Bielefeld University, Konsequenz 45, 33615 Bielefeld, Germany; 3 Department of Veterinary Biomedical Sciences, WCVM, University of Saskatchewan, 52 Campus Drive, Saskatoon, SK S7N 5B4 Canada

**Keywords:** Behavioural ecology, Evolutionary ecology

## Abstract

We require a better understanding of the relative contribution of different modes of non-genetic inheritance in behavioral trait development. Thus, we investigate variation in exploratory behavior, which is ecologically relevant and a target of selection. The metabolic hypothesis predicts exploratory behavior to be size-dependent across taxa. This size-dependency is cancelled out under high perceived risk, allowing us to determine the transgenerationally integrated estimated level of risk. Using fathead minnows *Pimephales promelas*, we manipulated perceived risk in mothers, fathers, caring males and offspring through continuous exposure to either conspecific alarm cues or to a control water treatment. In 1000 four-month old offspring, we determined body sizes and exploratory behavior. Perceived high risk in mothers, followed by personal risk, was most effective in eliminating size-dependent behavior whereas effects of paternal risk on offspring behavioral development were substantially weaker. When maternal risk is high, environmental mismatches between parents prevented offspring from responding appropriately to personal high risk. The environment of the caring male also impacted offspring behavior to a greater extent than that of its genetic parents. Our study highlights the high relative importance of maternal, personal and caring male risk environments and showcases potential costs of an environmental mismatch between parental sexes.

## Introduction

Phenotypic plasticity allows genotypes to express different phenotypes in different environments^1^. This ability is probably best-known from the strikingly deeper bodies that predator presence can induce in carp, to the extent that the same species was believed to be two different species for over 50 years^2^. Since selection acts on phenotypes, plasticity is a major force in evolution^3,4^. Interestingly, meta-analyses regularly find large differences in observable plasticity between studies^5,6^. This may be because considering plasticity as a within-generation phenomenon neglects the fact that plastic responses are the result from an integration of information from previous generations and personal information, which is also known as transgenerational plasticity^7–9^. One such instance are parental effects, which are known to be capable of adaptively priming offspring for living in the parental environment across taxa^10,11^. However, it remains unclear how different sources of transgenerationally transmitted environmental information, which may be conflicting with each other or with personal information, differ in their relative contribution to an individual’s phenotype^8,11^.

First, theory predicts that the relative priority of parental and personal environmental information crucially depends on the ontogenetic stage of individuals^12,13^. That is because juveniles need time to complete their sensory system development before they can accurately sample their environment^14^ and they need to sample their environment over a long time period to avoid effects of spontaneous environmental fluctuations. Thus, juvenile individuals should rely mostly on parental information. Only with increasing age, personal information should gain in relative relevance^15^.

Second, the parent that inhabits the same ecological niche as offspring and therefore repeatedly samples the same environment, should have a greater relevance on offspring development^8^. However, in anisogamic systems, these effects may be outweighed by the different modes of transgenerational transmission between parental sexes. While fathers can transmit environmental information only via epigenomes^16–18^, mothers can additionally embed nutrients, hormones, antibodies and enzymes within their large eggs^19,20^. That is likely why both the number of published studies as well as average effect sizes for maternal effects are larger than paternal ones^10^.

Third, receiving the same environmental information from different sources should increase reliability and predictive ability and thus have additive or synergistic effects^21,22^.

Fourth, as parental care impacts hypothalamic–pituitary–adrenal (HPA) axis development (analogous to the hypothalamic pituitary-interrenal (HPI) axis in fishes), involving corticotropin-releasing factor systems, it is suggested to be elementary in the development of behavior^23^ and in the transgenerational induction of altered behavioral phenotypes^24,25^. Unsurprisingly, multiple studies have suggested that parental care effects outweigh prenatal ones in juvenile animals^26,27^ but see Hellmann et al.^28^.

Clearly, we require more comprehensive studies that compare the relative importance of different information sources as well as their interactions in the transgenerational formation of phenotypes^8,11^.

To do so, we take advantage of predation, which represents one of the most common and forceful selection pressures in natural ecosystems^29^. Predation risk can impact prey demographics and cause cascading top-down effects on whole ecosystems through both direct removal of prey and indirect effects of fear^30,31^. The presence of predators as inferred by cues is known to induce distinct within-generation^32,33^ and transgenerational^11,34^ antipredator phenotypic plasticity, ranging from behavior and morphology to life-history. Antipredator plasticity is also known to be ontogeny-specific^35^. While the integration of information during the transgenerational response has already been comprehensively studied for group-based behavioral antipredator strategies in young juveniles^27^, we aim to investigate the relative importance of maternal, paternal, caring male and personal environments as well as their interactions in shaping individual behavior of the same species.

As a target individual-based behavior that is sensitive to predation risk, we selected exploratory behavior, (i.e., individual propensity to explore a novel environment), which is ecologically relevant and a target of selection^36^. We also take advantage of the metabolic hypothesis, which postulates that across taxa, the higher nutritional requirements of small individuals necessitate them to be bolder and more explorative than larger conspecifics, giving rise to size-dependent patterns^37^. While initial tests of this hypothesis could not exclude the possibility that the metabolic hypothesis emerges due to differences in age and experience^37,38^, it has since then been proven to be applicable even when standardizing age and experience levels^39^.

In the presence of predators, smaller individuals are also attacked and consumed more often, thus perceived predation risk plastically decreases exploratory behavior particularly in smaller individuals and thereby eliminates the size-dependent patterns that emerge from the metabolic hypothesis^39^. This well-established context allows us to accurately estimate the level of perceived risk that offspring integrate across conflicting or matching sources of information within and beyond generations. Using split-clutch designs, we manipulated perceived predation risk through regular exposure from birth onwards to either conspecific alarm cues (high-risk) or to a water control (no-risk). First, in the absence of parental care, we crossed this treatment across maternal, paternal and personal environments in a 2 × 2 × 2 design. Second, in the presence of parental care, we crossed risk treatment across biparental (gametic) and parental care environments in a 2 × 2 design; in total we thus had 12 treatment combinations. In addition, we also assessed average parental care intensity as a possible mechanism that communicates risk. In subadults, we then measured body sizes as well as emergence times from an isolation chamber, a proxy for exploratory behavior. We then analyzed our right-censored emergence data using three different statistical frameworks so as to verify the robustness of our results similar to Fraimout et al.^40^: with linear mixed-effect models (which are parametric, thus statistically efficient and allow the inclusion of random effects but whose distributional assumptions are violated by our censored data although this may be of low concern for large datasets such as ours), mixed-effect cox models (i.e., Kaplan–Meier estimators, which can consider right-censoring and allow the inclusion of random effects but due to their semi-parametric nature, they are of low statistical efficiency), and censored regression (Tobit) models (which are statistically efficient due to their parametric nature and can consider left- and right-censoring but do not allow the inclusion of random effects, which allows pseudoreplication to confound our results).

First, we expect paternal, maternal, caring parent and personal high risk to all eliminate patterns of size-dependent exploratory behavior, in line with previous research on the effects of personal high risk^37,39^. Second, as we sample subadults, we expect parental effects to outweigh personal environment effects. Consequently, we expect high risk in parents to eliminate size-dependent exploratory behavior to a greater extent compared to only personal experience with high risk. Third, as mothers but not fathers transmit hormones to offspring via their gametes, high-risk in mothers should outweigh paternal effects in the absence of parental care. The transmitted hormones should have similar consequences on offspring behavioral development as high personal risk, and thus eliminate size-dependent exploratory behavior. As receiving the same environmental information from different sources should increase reliability and predictive ability, we additionally expect additive or synergistic effects to emerge when more than one source of information in the parental generation provides high-risk information, which should generate stronger phenotypic responses than each of them in isolation. In contrast, as a previous study suggests the absence of additive effects between parental and personal information^41^, we hypothesize the same to be true in our study. Lastly, as parental care was suggested to be a crucial element in HPA/HPI axis development, we also expect parental care by high-risk males to eliminate size-dependent exploratory behavior, and this effect to be greater than that arising from gamete-mediated risk information.

## Results

**Figure 1:**
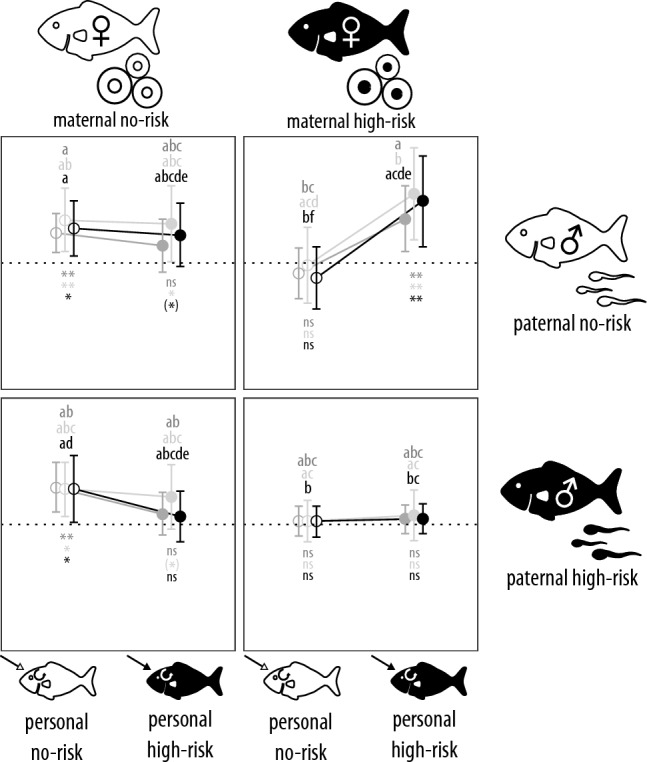
Relationship between exploratory behavior and body size (i.e., standardized regression coefficients (β) ± 95% confidence intervals) showcasing maternal × paternal × offspring risk interactions. The dotted line is the zero referent and represents size-*independent* exploratory behavior, positive scores indicate slower emergence (i.e., less exploratory behavior) with increasing body size; negative scores showcase the opposite. Mothers, fathers and offspring were exposed to either no-risk (white fish, empty dots) or high-risk (black fish, filled dots) environments. Different letters above bars indicate statistical differences between treatments across Figs. 1 and 3 at p < 0.05; asterisks below bars refer to the difference from zero (i.e., size-independent exploratory behavior). The different colors highlight the statistical methods used to derive effect sizes and p-values: mixed-effect cox models (light gray, estimated coefficients were multiplied with -1 for comparison purposes), linear mixed-effect models (dark gray) or censored regression models (black) controlling for family identity (all models) nested in tank identity (all except the censored regression model). **p < 0.01, *p < 0.05, (*) p < 0.1, ns p > 0.1.

**Figure 2:**
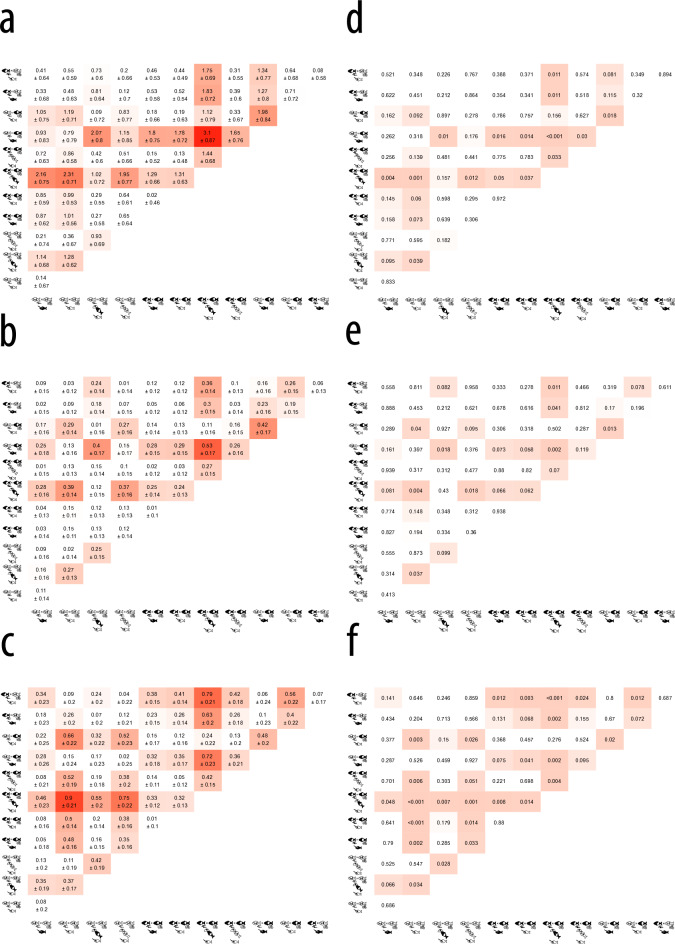
Pairwise comparisons between individual treatments shown as (**a**–**c**) estimated standardized regression coefficients β ± standard errors without leading signs to aid comparison, (**d**–**f**) p-values. Figures **a** and **d** show results from mixed-effect cox models, figs **b** and **e** results from linear mixed-effect models and **c** as well as **f** ones from censored regression (tobit) models controlling for family identity (all models) nested in tank identity (all except the censored regression model). Large effect sizes and small p-values are highlighted in red using scaled gradients. Schematic drawings along the axes of each figure showcase the respective risk treatment, with black fish indicating lifelong exposure to high risk, whereas white fish indicate exposure to a no-risk water control instead. More detail as to treatment identity is given in Fig. 4.

In the treatments that did not receive parental care, in two of the three analysis approaches, we found some evidence (i.e., p < 0.1) for a four-way interaction between body size, maternal, paternal and personal risk across different statistical approaches (Table 1). Post-hoc analyses suggest that in the absence of risk, patterns of exploratory behavior were size-dependent (Fig. 1). The presence of either maternal or personal high risk alone eliminated these patterns while this was not the case for paternal high risk alone (Fig. 1). Compared to high maternal risk, exposure to high personal risk caused lower estimated slopes across statistical approaches along with large variances in the mixed-effect cox framework, suggesting that effects of maternal high-risk slightly outweigh those of personal high risk. Surprisingly, when maternal high-risk was mismatched with paternal no-risk, offspring respond inappropriately to personal high-risk by displaying size-dependent patterns that are usually indicative for the absence of risk (Fig. 1); this mismatch led to the greatest effect sizes compared to the high-risk treatments across statistical approaches (Fig. 2). Cumulative maternal and paternal high risk also eliminated patterns of size-dependent exploratory behavior, but at the same time also slightly decreased variances (Fig. 1), which also led to large effect sizes compared to no-risk treatments (Fig. 2); there was no further cumulative effect by the addition of personal high risk. Likewise, there was no evidence for the combination of personal and paternal high-risk environments inducing cumulative effects (Fig. 1).

**Table 1:** Full models analyzing effects of risk treatments across generations and parental care intensity on 123-day old *Pimephales promelas* exploratory behavior.

	lme4			coxme			censReg		
	df	χ^2^	p	df	χ^2^	p	df	χ^2^	p
**Parental care absent**									
Body size	1	0.884	0.347	1	0.464	0.496	1	0.160	0.689
Maternal risk	1	1.309	0.253	1	0.115	0.735	1	4.168	**0.041**
Paternal risk	1	0.677	0.411	1	0.755	0.385	1	4.046	**0.044**
Personal risk	1	0.071	0.789	1	0.165	0.685	1	0.062	0.804
Body size × maternal risk	1	0.293	0.588	1	0.951	0.330	1	1.841	0.175
Body size × paternal risk	1	3.571	*0.059*	1	5.369	**0.021**	1	2.593	0.107
Maternal risk × paternal risk	1	0.992	0.319	1	0.519	0.471	1	2.879	*0.090*
Body size × personal risk	1	0.036	0.849	1	0.018	0.893	1	0.044	0.834
Maternal risk × personal risk	1	0.007	0.932	1	1.134	0.287	1	0.001	0.980
Paternal risk × personal risk	1	0.002	0.969	1	0.176	0.675	1	0.013	0.909
Body size × maternal risk × paternal risk	1	2.043	0.153	1	1.988	0.159	1	3.058	*0.080*
Body size × maternal risk × personal risk	1	0.269	0.604	1	0.001	0.971	1	0.630	0.427
Body size × paternal risk × personal risk	1	4.084	**0.043**	1	3.562	*0.059*	1	2.733	*0.098*
Maternal risk × paternal risk × personal risk	1	0.788	0.375	1	0.230	0.632	1	0.573	0.449
Body size × maternal risk × paternal risk × personal risk	1	2.765	*0.096*	1	2.357	0.125	1	3.549	*0.060*
**Parental care present**									
Body size	1	2.711	0.100	1	3.563	*0.059*	1	5.28	**0.022**
Caring parent risk	1	0.32	0.572	1	0.167	0.683	1	0.731	0.393
Biparental (gametal) risk	1	0.238	0.625	1	0.096	0.757	1	0.394	0.53
Parental care intensity	1	0.14	0.708	1	0.139	0.709	1	0.102	0.749
Body size × caring parent risk	1	4.53	**0.033**	1	4.832	**0.028**	1	7.261	**0.007**
Body size × biparental (gametal) risk	1	0.98	0.322	1	2.323	0.127	1	3.189	*0.074*
Biparental (gametal) risk × caring parent risk	1	0.11	0.740	1	0.141	0.707	1	0.34	0.56
Body size × biparental (gametal) risk × caring parent risk	1	0.037	0.847	1	0.269	0.604	1	0.326	0.568
**Post-hoc: overall treatment effects**									
Body size	1	1.093	0.296	1	5.195	**0.023**	1	0.368	0.544
Risk treatment	11	6.461	0.841	11	7.300	0.774	11	27.046	**0.005**
Body size × risk treatment	11	19.35	*0.055*	11	21.992	**0.024**	11	39.282	** < 0.001**

Shown are the results from three different analysis approaches: linear mixed-effect models (R package lme4), mixed-effect cox models (R package coxme) and censored regression models (R package censReg). In the first two approaches, family identity nested into tank identity were included as random effects. However, censReg does not allow the presence of random factors and did not compute when these factors were added as fixed factors instead, thus with these models, we could not control for family or tank identity. Significant effects and interactions (p < 0.05) are highlighted in bold font, and tendential ones (p < 0.1) in italics.

**Figure 3:**
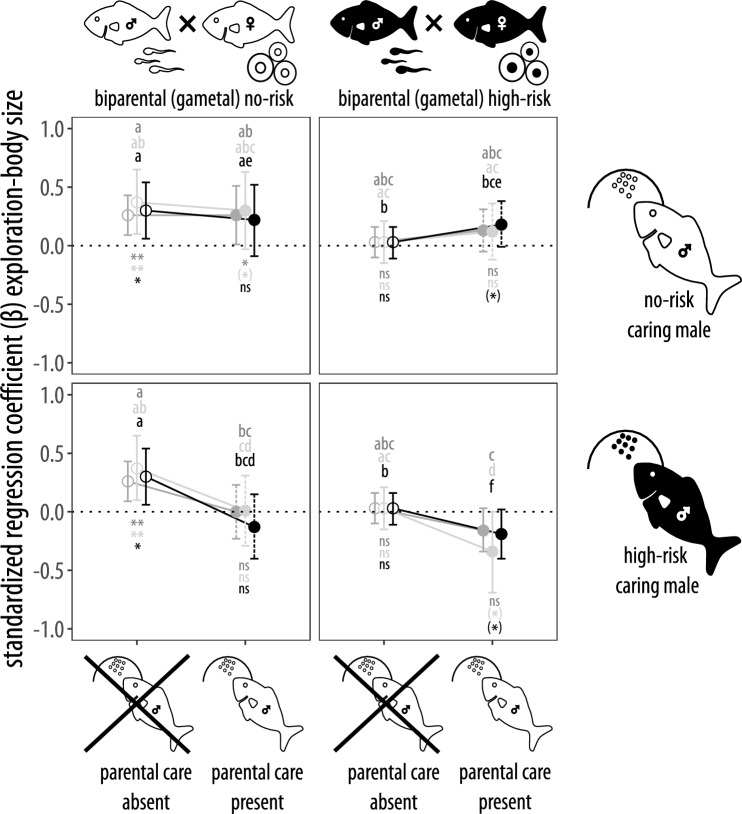
Relationship between exploratory behavior and body size (i.e., estimated standardized regression coefficients (β) ± 95% confidence intervals) showcasing biparental risk × caring male risk × presence of parental care interactions. The dotted line is the zero referent and represents size-*independent* exploratory behavior, positive scores indicate slower emergence (i.e., less exploratory behavior) with increasing body size; negative scores showcase the opposite. Genetic parents and caring males were exposed to either no-risk (white fish) or high-risk (black fish) environments while personal risk was consistently low. Parental care was either absent (empty dots) or present (filled dots). Dashed error bars highlight treatments that involved cross-fostering. Different letters above bars indicate statistical differences between treatments across Fig. 1 and 3 at p < 0.05; asterisks below bars refer to the difference from zero (i.e., size-independent exploratory behavior). The different colors highlight the statistical methods used to derive effect sizes and p-values: mixed-effect cox models (light gray, estimated coefficients were multiplied with -1 for comparison purposes), linear mixed-effect models (dark gray) or censored regression models (black) controlling for family identity (all models) nested in tank identity (all except the censored regression model). **p < 0.01, *p < 0.05, (*) p < 0.1, ns p > 0.1.

Analyzing the exploratory behavior of individuals that received parental care revealed a strong interaction between body size and caring parent risk whereas there was no overall main effect of biparental (gametal) risk or a further interactive effect of this term across statistical approaches (Table 1). Average parental care intensity also did not impact patterns of exploratory behavior. Post-hoc analyses confirm these results by showing that in the presence of parental care, biparental high risk alone did not cause a strong shift away from size-dependent exploratory behavior whereas the presence of a high-risk caring male alone clearly did (Fig. 3), this also becomes clear when looking at the large effect sizes of this treatment compared to the other ones (Fig. 2). Interestingly, in offspring from high-risk parents, the presence of a no-risk caring male was able to shift phenotypes slightly in the direction of size-dependent exploratory behavior compared to when no caring parent was present (Fig. 3). Finally, there was weak evidence for cumulative effects of biparental high risk and caring parent risk, as despite large effect size differences to many other treatments (Fig. 2), large variances particularly in the mixed-effect cox framework make it unclear whether exploratory behavior was independent of body size or whether we have evidence for *negative* size-dependent exploratory behavior (Fig. 3).

## Discussion

Our results showcase the relative contribution of different modes of non-genetic inheritance on the development of offspring exploratory behavior, which is ecologically relevant and a target of selection^36^. As expected, patterns of exploratory behavior were consistent with the metabolic hypothesis (i.e., size-dependent) when risk was coherently low across parental and personal environments (Fig. 1 top left panel). Furthermore, in accordance with previous studies, personal high-risk environments alone were sufficient to decrease exploratory behavior in smaller individuals, which eliminates size-dependent patterns and constitutes a typical high-risk phenotype (Fig. 1, top left panel). This effect was even stronger when only high maternal risk was present, but paternal risk alone did not impact behavioral patterns (Fig. 1, bottom left panel). Interestingly, under maternal high-risk and paternal no-risk, which constitutes a mismatch between the risk environments of the gametic parents, offspring capacity to develop high-risk phenotypes in the presence of high personal risk appears to be impeded (Fig. 1, top right panel). In the presence of parental care, effects of the risk environment in the caring parents clearly outweighed the experience that gametic parents had with high risk (Fig. 3), with a high-risk male alone being sufficient to induce high-risk phenotypes alone (Fig. 3 bottom left panel) and no-risk caring males being able to almost outweigh the effects of high biparental risk (Fig. 3 top right panel).

That high perceived predation risk can eliminate size-dependent patterns of exploratory behavior is consistent with previous research^37–39^. This is likely because plasticity-induced decreased exploratory behavior is advantageous in a predation context, where reduced boldness and exploration is linked to higher survival^42^. On the other hand, plasticity-induced decreased exploratory behavior may also be costly in certain contexts as it is also associated with lower dispersal propensity^43^, which reduces population spread, increases inbreeding and thereby leads to a lower effective population size due to the Allee effect^44^. However, when dispersal is risky, for example when a predator-free environment is surrounded by ones containing predators, low dispersal may be beneficial. Furthermore, consequences for community dynamics and ecosystems are likely as well: similar to how decreased boldness relaxes selection pressures on lower trophic levels^45^, lower exploratory behavior may likewise lead to a trophic cascade within and between communities. In addition, the reduced “landscape of fear” that results from decreased exploratory behavior across populations also indirectly increases fecundity of lower trophic levels, which causes cascading top-down effects across food webs, thereby increasing selection pressure particularly on primary producers^30,31,46^.

Maternal high risk impacted offspring phenotypes to a greater extent than personal experience with high risk. This is in accordance with theory predicting that the relative priority of parental and personal environmental information crucially depends on the ontogenetic stage of individuals^12,13^. Offspring can only accurately sample their environment after their sensory systems have fully developed^14^. However, to minimize the impact of spontaneous environmental fluctuation on their environmental assessment, they need to sample the environment repeatedly over time. Thus, parental information remains highly relevant during early ontogeny as shown in sheepshead minnows *Cyprinodon variegatus* where parental temperature effects were greatest at an early age^47^; only with increasing age, the impact of parental information is devalued^15^. We tested individuals as 123-day old subadults, and *P. promelas* only completes their larval development at ~ 18 days age^48^, giving them an effective sampling period of 105 days. In contrast, their parents deposited clutches after having an effective sampling period of between 159 and 328 days, which makes that parental environmental assessment likely more robust compared to personal environmental assessment.

Maternal risk environment effects on offspring exploratory behavior outweighing paternal ones in the present study is also in accordance with previous studies that highlight the larger relevance of maternal effects during transgenerational responses^10^ and suggests the presence of sex-specific transgenerational plasticity^8^. This may sometimes arise due to differences in information reliability between parents^8^. However, being the sex that provides parental care, male *P. promelas* are more likely to share their environment with offspring than females, which should make paternal environmental information more reliable^49^. Thus, the observed maternal effect is likely because mothers have more mechanisms available to transmit information over generations, *e.g.* the ability to deposit hormones within eggs^19^ whose levels directly influence behavioral phenotypes^50,51^. Fathers can only transmit epigenomes via sperm^16–18^, thus their only opportunity to transmit hormones to offspring is during parental care (see below).

Environmental mismatches between maternal and paternal risk information when maternal risk was high emerged to be costly as it hindered offspring in responding appropriately to personal high-risk. Mismatches between parental risk environments are also associated with lower survival in the presence of predators^52^. When both parents are exposed to the same environment, which is a common design in transgenerational plasticity research, such costs are hidden. They might also emerge outside of a predation context as long as fathers and mothers have experienced different environments prior to mating. A potential proximate mechanism underlying this observation is that the epigenomes that fathers transmit through sperm might alter offspring sensitivity to maternal hormones embedded within eggs but this hypothesis remains to be confirmed by future research. While such environmental mismatches are rather unlikely to occur in nature for our model system as it is a non-migrating species and mostly spreads through failed bird predation attempts and occasional spring floods, it is much more likely to occur in nature for mobile species and populations with expanded ranges, such as migrating birds or fish. An example are stickleback *G. aculeatus*, where migrating anadromous fish (i.e., those originating from a marine high-risk environment) might hybridize with no-risk freshwater conspecifics and thereby generate inferior hybrid offspring^53^. Such costs of plasticity may also be the reason that cosmopolitan plastic generalists are often outperformed by locally adapted specialists^54^. Thus, our finding contributes to our understanding of the complex relationship between plasticity and local adaptation^55^. On a more general scale, such costs may also represent an additional barrier to hybridisation and may thus contribute to species isolation, as has recently been showcased in molly hybrids, which are sub-optimally adapted to their parental environments^56^.

Additive effects from father-offspring and mother-father environmental matching beyond a minor influence on variances when both mothers and fathers had the same environmental experience were not observable. Although this result contradicts another study of *P. promelas* that found an strong additive effect between high parental and high personal risk on freezing behavior^57^, it is in line with the non-additive effects that were found in snails^41^ and sticklebacks^58^. Differences between studies may emerge because the interaction between parental and personal environments is usually trait-dependent^59^. This is because traits differ in their capacity to reach optimal expression levels when information is provided from only a single environment^60^. Another alternative explanation is that as we simulated constant presence or absence of predation risk in our study, the environmental grain was too coarse to reveal additive effects, which may only emerge when perceived risk levels fluctuate more and this might be another exciting avenue for follow-up research.

The risk environment of the caring males affected offspring exploratory behavior to a greater extent than gametic parent risk environments, in accordance with previous studies suggesting that parental care effects outweigh prenatal ones^26,27^ and that parental care has an elementary role in the transgenerational induction of behaviors^24,25,61^, to the extent that environmental change during the parental care period itself is sufficient to induce phenotypic change in offspring^62^. Surprisingly, differences in parental care intensity were not directly correlated with exploratory behavior, which contrasts rat studies suggesting this to be the case^24,25^. While this might be a side-effect of us being unable to track the exact frequency of individual parental care behaviors such as egg rubbing or nibbling due to the low quality of the video footage, it might also suggest that in our case, exploratory behavior may be influenced by alternative mechanisms during parental care. The capability of fish embryos to recognize and differentiate between risk cues from within their eggs^63,64^ allows caring males to modify offspring personalities through the release of disturbance cues^65^, gill-released steroid hormones^66^ or through mechanosensory cues^67^, following the hypothesis that these cues mimic parental environments^68^. Nevertheless, future studies may benefit from using high-speed and high-resolution cameras to track individual parental care behaviors as doing so may allow us deeper insight into the mechanisms by which parental care modulates the behavioral plasticity of *P. promelas* offspring.

Overall, while our effect sizes appear to be rather small (average estimated difference across statistically significant differences between treatment pairs; lme4: 0.371 ± 0.152; coxme: 1.848 ± 0.732; censReg: 0.506 ± 0.184), they still outweigh effect sizes from our previous research on the effects of personal risk on the same relationship between body size and exploratory behavior^39^ where, when applying the same statistical frameworks as here, estimated differences between high and low personal risk ± SE were: lme4: 0.354 ± 0.160; coxme: 0.320 ± 0.212; censReg: 0.420 ± 0.200. Furthermore, our sample sizes are rather large, and power analyses suggest that at an α of 0.05, we had 80% power to observe effects within the range of d_Cohen_ = 0.238–0.330 (i.e., small effects) between treatment pairs. Of course, the true effects may be even smaller, in line with recent meta-analyses suggesting that evidence for anticipatory parental effects is rather weak^69,70^. If this is the case, our sample sizes may be too low to detect such small effects. Alternatively, the absence of effects in our studies could also be Type II errors. Another explanation is that the commercial laboratory *P. promelas* population that we had to use for the present study (as wild-caught individuals failed to reproduce in the lab) may suffer from some degree of inbreeding despite being outcrossed in regular intervals, which can reduce observable plasticity^71^. While one may also argue that the long time in the laboratory has selected individuals to maximize their fitness in a no-risk environment as costs of plasticity may select against its maintenance, meta-analyses suggest that costs of plasticity are surprisingly low to non-existent^72^, and our *P. promelas* population shows clear evidence for both within-generation^39,73–75^ and transgenerational antipredator plasticity^27,57^. Likewise, despite selective breeding in artificial habitats, goldfish *Carassius auratus* have retained their antipredator plasticity for over 3000 years, likely due to the power of selection by predation risk^76,77^. On the upside, our use of laboratory populations also helps avoiding confounding issues with unknown pathogen infection^78^ which likewise can limit observable plasticity^79^.

Another limitation of our study is that due to the lack of simultaneously spawning pairs we could only set up two of the four parental treatments as cross-fostering treatments although it would have been ideal to do so for all treatments that involved parental care. Male *P. promelas* readily adopt unrelated eggs as caring for eggs increases their attractivity to females^80^. On the other hand, they do provide less care to unrelated eggs in general^81^ and in our study in specific^27^. However, we included parental care intensity in our models to control for the possibility that differences in parental care levels impact our results. In addition, effects of no-risk parental care and high-risk parental care were surprisingly similar independent of males caring for own offspring or adopted ones (Fig. 3) and parental care by high-risk and no-risk males had opposite effects on offspring exploratory behavior independent of whether they provided care for own or adopted clutches (Fig. 3). This suggests that our results are unlikely to be affected by this shortcoming of our experimental design.

Our results showcase the relative contribution of different modes of non-genetic inheritance in the development of exploratory behavior. However, we still do not know to what extent the observed patterns here are generalizable, which requires more equally comprehensive studies on how the integration of information within and across generations affects behavioral development in other traits, environments and taxa. Within such studies, behavioral variation across ages and sexes should also be considered since these dimensions of phenotypic variation are being increasingly recognized across plasticity studies^35,75^. For example, assessing whether plastic changes in behavior emerge mainly in one offspring sex can be done even at early life-stages using genetic approaches. This knowledge is also of ecological relevance since sex-specific plasticity can drive either the evolution of assortative or disassortative mating systems and modulate sexual conflict intensity^44^. Lastly, future studies should also consider sampling individuals repeatedly, which we could not do due to time constraints, as this would allow us to gain insight into how transgenerational plasticity impacts animal personality.

## Methods

### Ethical note

The present study was planned in accordance with the Animal Research: Reporting of In Vivo Experiments (ARRIVE) guidelines, complied with Canadian laws, including the Canadian Council on Animal Care (CCAC) guidelines for humane animal use, and was approved by the University of Saskatchewan’s Animal Research Ethics Board (Animal Use Protocol: 20170089).

### Experimental fish

Our model species is the fathead minnow *P. promelas*^82^, a small cyprinid fish widespread across Northern American freshwaters^49^. After forming shoals as juveniles, adult males become solitary, territorial and provide alloparental care to clutches while females remain shoaling and do not contribute to parental care after spawning^80^. Fathead minnow populations experience fluctuating predation risk^73^, which induces both within-generation^39,73–75^ and transgenerational antipredator plasticity^27,57^. Like in other taxa, in this species, exploratory behavior is known to be size-dependent when individuals grow up under no-risk but not under high-risk conditions^39^.

**Figure 4:**
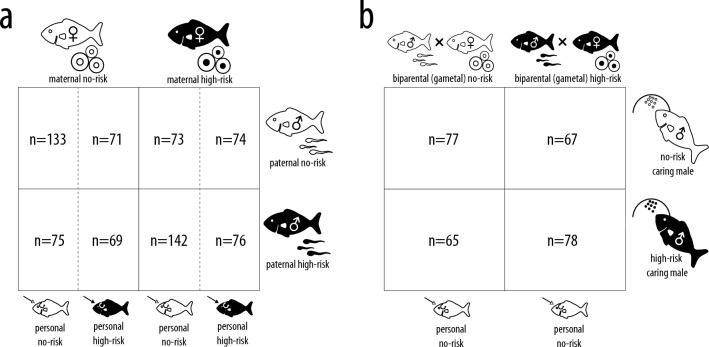
(**a**) 2 × 2 × 2 breeding design aiming to capture individual and cumulative effects of paternal, maternal and personal risk on individual morphology in the absence of parental care. (**b**) 2 × 2 breeding design aiming to capture individual and cumulative effects of biparental (gametic) risk and caring male risk on individual morphology. Sample sizes of individuals whose behavior was analysed are stated at each treatment intersection.

As experimental animals, we used fish that were unfamiliar with predators as they were derived from a laboratory stock population that were removed from the wild (unknown founder population size) and housed by a commercial fish supplier for over 35 years (i.e., 17–35 generations) while being outcrossed with individuals from other commercial fish hatcheries and research laboratories in 1989, 1993, 1999, 2005 and 2017. While it may appear that such selection to a no-risk environment may eliminate antipredator plasticity, previous studies on fish from the same population^27,39,74,75^ as well as on *Carassius auratus*, a species that had been bred in captivity for over 3000 years^76,77^ suggest that antipredator phenotypic plasticity is retained even after such long periods, likely because predation is a strong selection pressure. These fish were then raised from hatching onwards under continuous exposure to either alarm cues (3.302 × 10^–6^ cm^2^ conspecific skin/l five times a week, an innately recognized, reliable signal of high predation risk^65^ that also induces different exploratory behavior^39^ or to a no-risk water control. As opposed to predator kairomones, to which fish habituate after repeated exposure, no habituation was shown to occur even after repeated exposure to conspecific alarm cues without other evidence of a predatory threat in sea lamprey^83^, in cichlids^35,84,85^ and in fathead minnows^27,57,74,75^. We then crossed this risk treatment across maternal, paternal and personal environments in the absence of parental care to generate a full-factorial 2 × 2 × 2 design. Then, to assess the relative importance of parental care, we used parts of the same clutches to set up another 2 × 2 design that crossed (genetic) biparental risk with different risk levels experienced by caring males; care was performed by either genetic (own) fathers (whenever biparental risk matched caring male risk) or by foster males of the opposing risk treatment (whenever biparental risk did not match caring male risk) and offspring were consistently raised in no-risk environments. Taken together, this resulted in 12 different risk treatments (Fig. 4).

In the parental individuals, the above-described risk treatment impacted exploratory behavior^39^, shoaling behavior^74^ and morphology^75^; during these assays, risk levels were not modified and fish from different risk treatments were not handled differently. To generate offspring, at 7–13 months age, we paired high-risk and no-risk parental individuals from different families (so as to avoid inbreeding) in all four possible parental treatment combinations (Fig. 4) and bred them within 10-l tanks containing gravel, a gently bubbling airstone and two breeding tiles each. Every day, pairs received 35% water changes and ad libitum feedings with frozen *Chironomus* sp. bloodworms to facilitate egg production. Within breeding tanks, risk treatments were discontinued to avoid exposure of embryos to residual cues; eggs were laid within a median of 4 days after individuals were paired up (interquartile range: 2–9, range: 0–24). Upon egg deposition, we removed tiles from tanks and gently rubbed eggs off using a moist rubber glove. For the treatments involving parental care, we only removed half of the eggs in this way and then returned the tile on top of a Ø 10 cm petri dish that was covered with a 3 × 3 mm plastic mesh to avoid the common cannibalization of freshly hatched juveniles, which may release alarm cues, in this species; this mesh also prevents further parental care after hatching while chemical cues could pass. Additionally, as soon as we observed the start of hatching, we removed the tile from the tank and let eggs hatch in a separate tank. After hatching, fry were split into full-sib groups each containing 10 individuals, and groups were randomly assigned to either the high- or low- risk treatment. To raise fry, we matched tank size, water change frequency and food regimen to developmental stages to conform to the increasing space requirements of growing fish. From day 1–38, they were raised in 500 ml tanks, received 80% water change every day and were fed daily with live *Artemia* nauplii (1-3d: 1 µl/fish, 4-18d: 10 µl/fish, 18-38d: 20 µl/fish). At day 39, we recorded offspring shoaling behavior and found that shoaling density patterns were mainly driven by the risk environment of caring males^27^. Subsequently, fish were transferred into 10-l tanks where they received weekly 25% water changes and were fed daily ad libitum with flake food (Nutrafin A6840 Max Goldfish Flakes, Hagen, Mansfield, MA, U.S.A). Light was consistently provided through white (5500 K) LED strips directly above tanks in a 16:8 light:dark cycle (6am — 10 pm). More details are provided in the Supplementary Information 3. At 123 days age, we assayed the exploratory behavior of each individual.

### Parental care

In the four risk treatments that involved parental care, we assessed parental care intensity from the 22 caring males by filming (C922x Pro Stream, Logitech, Suzhou, China) them daily for 10 min from day 0 to day 3 clutch age, in total four times (details in in the Supplementary Information 3). In total, we recorded 164 care videos of 41 clutches that were being cared for.

### Exploratory behavior assays

At 123 days of age, we measured emergence from an isolation chamber as a proxy for individual exploratory behavior as described in Meuthen et al.^39^ and in the Supplementary Information 3. In the past, such emergence times have been used to infer boldness^39,86^, but they are now established as a measure of exploratory behavior^36,40^. In brief, we moved individuals into 26 × 50 × 30 cm (L × W × H) assay tanks containing 13 l of water (20 ± 0.1 °C) and a breeding tile in the center as well as a Ø10 cm isolation chamber at one end that was initially closed but could be opened during the trial. After transferring individual fish into the chamber, we let them acclimate for 20 min, followed by a 20 min emergence period, during which fish were filmed (C922x Pro Stream). Afterwards, we assessed body size (standard length, i.e., from the tip of the snout to the caudal peduncle) to the nearest millimeter. We chose to not present food in our assay following previous assessments of size-dependent exploratory behavior across taxa^37–39^, although doing so may have increased the benefits of being explorative and thereby facilitate overall exploratory behavior. That is because the presence of specific food items together with variable individual preferences for these items may skew observed emergence patterns. Furthermore, in natural environments, individuals are often unaware of the presence of food outside of their shelter unless they emerge and explore that environment. Lastly, the metabolic hypothesis predicts that independent of food availability, small individuals will always have higher nutritional requirements than larger conspecifics, and patterns of size-dependent exploratory behavior should remain. In total, we tested 1100 minnows from 69 different clutches but 100 trials had to be excluded due to technical failures or unexpected disturbances. Thus, we analyzed 1000 fish (65–142 individuals per risk treatment, see Fig. 4). Because we measured each individual only once, we cannot conclude anything on the existence or formation of the personality trait exploration, and instead focus our discussion on the factors that explain variation in exploratory behavior.

### Data analysis

#### Parental care

Care behavior was assessed during the last five minutes of each video in order to standardize the time after camera placement (i.e., visual disturbance). Initial screening revealed that the quality of the video footage was insufficient to reliably track high-frequency parental care behaviors such as egg rubbing and nibbling. However, as these behaviors were usually performed constantly as long as the caring male was in the vicinity of the eggs (D.M., personal observation), as a proxy for parental care intensity, we measured the proportion of time that the caring male spent within one standard length distance of the clutch, i.e., the time it spent inside the breeding tile to which the eggs were adhered^87^. In total, we analyzed 160 recordings from 20 males caring for 40 clutches, from which 280 offspring were tested in exploratory behavior assays (Fig. 4). Analyzing this data, Meuthen et al.^27^ found that variation in parental care intensity is not directly driven by risk treatment but instead by the day of care (~ 10% increase over the 4 days), the proportional change in clutch size caused by removing or swapping eggs (up to 40% differences in care intensity) and by whether males cared for own or adopted eggs (~ 12.4% more care for own eggs). Here we focus on investigating whether parental care intensity modified exploratory behavior beyond risk treatments; for every clutch we thus calculated average parental care intensity over the 4-day period.

#### Exploratory behavior assays

Exploration was assessed as the latency to emerge with the entire body from the isolation chamber (in seconds) after it was opened. If an individual did not emerge within 20 min, it was assigned a threshold value of 1200 s.

### Statistical analysis

All analyses were conducted with R 4.3.0. Because our exploratory behavior data was right-censored due to the 1200 s threshold value, some researchers suggest it not to be suitable for classical linear regression approaches, see e.g. Edelaar et al.^88^. Thus, similar to the approach of Fraimout et al.^40^, we followed three different statistical frameworks to verify the robustness of our results. To allow comparison between frameworks, we first transformed our continuous variables (emergence time, body size, average parental care intensity) into z-scores using the scale function from the base R package.

First, as with large sample sizes, linear mixed effects models are also suggested to be robust to violations of distributional assumptions^89^, we fit them using the lme4 R package v1.1–33^90^. As random effects we entered family identity (to control for genetic effects) nested in tank identity (to control for tank effects).

Second, we used mixed-effect cox models, which adopt the Kaplan–Meier survival analysis framework, and fit survival curves using the coxme R package v2.2–18.1^91^. Because the event measured in our time-to-event data is emergence from the shelter rather than actual death or survival as is usually the case in such analyses, the estimated survival curves correspond to the expected proportion of fish having emerged from the refuge at a certain time. Here as well, as random effects we entered family identity nested in tank identity.

Third, we fitted censored regressions (Tobit models) using the censReg R package v0.5–36^92^. We set the right limit for censoring at 1.652215129 (the z-score corresponding to the maximum time value in seconds in our experiment) and the left limit at – 1.000734669 (the z-score corresponding to zero in our experiments). As censored regression models do not allow the specification of random effects and require each fixed effect to be present across other fixed effects, we had to run full models without including family or tank identity. However, for analyses that concerned individual treatments or pairwise comparisons (i.e., models without a treatment variable or those where families were shared across treatments), we entered family identity as a covariate to control for genetic effects. Tank identity could never be added to censored regression models as it was never shared across other fixed factors.

Across approaches, we aimed to analyse the correlation between emergence times and body sizes in accordance with the metabolic hypothesis and how this correlation is affected by the different risk treatments. First, we split our dataset into the treatments that received parental care and those that did not. For the individuals receiving no parental care, we entered body size, maternal risk, paternal risk, personal risk and all 2-way, 3-way and 4-way interactions between these factors in the model. For the dataset containing individuals receiving parental care instead, we entered body size, biparental (gametal) risk and caring parent risk along with all 2-way and 3-way interactions in the model. In addition, to control for possible effects of different parental care intensity levels, we entered average parental care intensity as a covariate in the model but did not include interactions involving this term as our number of samples that received below 50% parental care intensity was too low to obtain reliable interaction estimates (e.g. in two treatments no pairs provided such little care, and in the other two treatments this was the case only for 1–2 pairs each). Second, as full models suggest the presence of higher-order interactions throughout, we followed up with a simple post-hoc model where we only entered body size, a single ‘treatment’ fixed effect that contains all 12 treatment combinations as well as their interaction. This approach also mitigates the risk of overfitting models^93^. We tested for the significance of the fixed effects in all models using Wald chi-square tests using the Anova function in the car R package. Afterwards, we extracted pairwise contrasts between treatments from the full model using the emmeans R package v. 1.8.0^94^ or, in case of CensReg models, which are not supported by emmeans, we split the dataset in all possible pairwise comparisons and re-ran the models to derive contrast estimates. Lastly, we split our dataset into 12 datasets, each containing only the data from a single treatment so as to re-run all models without a ‘treatment’ fixed effect so as to calculate estimated slopes (β), their variation, and whether they are significantly different from zero. All data (Supplementary Information 1) and corresponding metadata (Supplementary Information 2) that were used in statistical analyses are provided as part of the supplementary material.

### Supplementary Information

Supplementary Information 1.

Supplementary Information 2.

Supplementary Information 3.

## Data Availability

Data are provided as part of the supplementary material.
