# Supplementary material for: Transgenerational plasticity of exploratory behavior and a hidden cost of mismatched risk environments between parental sexes
Source: Sci Rep. 2023 Nov 13;13:19737. doi: 10.1038/s41598-023-46269-8 (PMC10643415; doi:10.1038/s41598-023-46269-8)
Supplement: Supplementary file 3 — Supplementary Information 3. [file 41598_2023_46269_MOESM3_ESM.pdf]

Transgenerational plasticity of exploratory behavior and a hidden cost of mismatched risk environments between parental sexes

Denis Meuthen, Arash Salahinejad, Douglas P. Chivers, and Maud C. O. Ferrari

Supporting Information

1. Experimental fish

To set up our experimental design (Fig. 4 of the main manuscript), we derived 81 fish (40 males and 41 females) from our laboratory stock population. Parental fish were lifelong subject to either perceived high (20 males, 20 females) or no risk (20 males, 21 females). The 81 fish used here were 7-13 months old and sexually mature with males displaying their typical black and white nuptial coloration whereas females had developed a pronounced belly area. We removed these fish from their stock tanks (that each housed different families) and formed outbred pairs according to our experimental design (Fig. 4 of the main manuscript) by moving them together into 34.5 x 27.0 x 18.5 cm (L x W x H) tanks (PC90 10l with LID90I-4 blue poly lid, Pentair Aquatic Eco-Systems, Atlanta, USA). Each of these tanks contained 375 ml of gravel (mean±SD weight: 540 ± 15 g), a gently bubbling airstone and two breeding tiles (halved PVC pipes, ⌀ 9.5 cm, length 7.5 cm). Tank temperature varied seasonally but not between treatments (median 24.8 °C, IQR 2.1 °C, range 16.5 – 29.8 °C; within-tank variation: IQR 2.2 °C , across-tank variation: IQR 0.475 °C) and light was provided in a 16:8 light:dark cycle (6am — 10 pm). The four different parental

treatment combinations were always set up at the same time so that different treatments never differed in parental age or holding conditions. In total, we formed 44 pairs, 10-12 pairs per parental treatment combination. Every day, at 11 am and 5 pm, we visually checked tiles if any eggs were attached to them; if that was the case, we removed the tile with the help of a plastic bin so as to keep eggs underwater at all times. With a moist rubber glove, we then gently rubbed eggs off the tile; in all treatments that involved parental care, we removed only approximately half of the clutch and returned the remaining eggs to the caring parents. In the two cross-fostering parental care treatments (Fig. 4 of the main manuscript), as long as the pair that was to provide parental care laid an own clutch within ±12 hours, we swapped in the tile with the unrelated eggs immediately after removing a part of the clutch. Between the four parental care treatments, there was no significant difference in how many eggs they cared for (Kruskal-Wallis test, χ^2=6.425, df=3, p=0.093) or in the proportion of how many eggs the clutch was altered by through removing eggs or swapping tiles (Kruskal-Wallis test, χ^2=2.481, df=3, p=0.479). Eggs were then transferred into Ø 12 cm plastic cups (each containing a gently bubbling airstone, 500 ml water) with constant temperatures across cups and treatments that fluctuated seasonally: within-tank variance: median 22.9 °C, IQR 1.9 °C, range 18.1 – 26.2 °C), 80% of the water volume was replaced with fresh water daily. When fry hatched from the eggs, dependent on fry availability, we split them into two to three replicates (10 fry each) per personal risk treatment. Additional fry (median 25.5 fry, IQR 37.75 fry, range 0 –

237 fry) were moved into 20 x 30 x 12.5 cm (L x W x H) tanks containing 5l of water so as to generate a density-unmatched back-up replicate which was otherwise treated the same as the other replicates. If any fry in the main replicates died during the first 39 days after hatching, dead fish were replaced with a random fish of the respective back-up replicate. Mortality up to this age did not differ significantly between treatments (median 0 %, IQR 9.1 %, range 0 – 60 %; Kruskal-Wallis test: $\chi^2 = 10.122$, $p = 0.519$). At 39 days age, we then transferred the groups of 10 fish each into 34.5 x 27.0 x 18.5 cm tanks that were set up the same as the tanks used for breeding (375 ml gravel, airstone, two breeding tiles). Within these tanks, temperature varied seasonally (median 22.3 °C, IQR 4 °C, range 11.1 – 29.3 °C) but as different treatments were always in direct vicinity to each other, there was no difference in temperature between treatments. Different tanks were consistently visually separated by white opaque plastic sheets and chemical cues were never mixed between tanks.

2. Recording parental care

We assessed parental care intensity by videotaping caring males daily for 10 minutes from day 0 to day 3 (in total 4 times) clutch age, as fry consistently hatched on the 4th day. For this purpose, between 6 – 8 pm, we placed a web camera (C922x Pro Stream, Logitech, Suzhou, China) that was mounted on top of a tripod 15 cm in front of the tank. Videos were always recorded prior to the second feeding of the day to ensure that residual food did not confound parental behaviour. Furthermore, on day

0, we recorded caring parent behaviour only 2 – 4 hours after we returned or swapped the tile with the clutch so as to ensure that parents resumed normal brood care activities.

3. Exploratory behavior assays

At 123 days of age, individual minnows were assessed for exploratory behavior using emergence trials as described in Meuthen, et al.[1]. The tanks used for the emergence trials were 26 x 50 x 30 cm (L x W x H) in size, filled with 13 l water (temperature 20 ± 0.1 °C), and each tank contained a Ø 10 cm isolation chamber at one end that was initially closed but could be opened by the experimenter during the trial. Furthermore, the tank contained a breeding tile at a distance of 15 cm from the isolation chamber so as to facilitate emergence. To avoid temperature differences between holding tanks and experimental tanks confounding our results, we always moved tanks containing the experimental fish into the experimental room one day prior to testing so that they were acclimated to experimental temperatures. On the day of the experiment, minnows were caught with a net from their holding tank, transferred indiviually inside the isolation chamber, whose top was then closed off with a tile. After a 20 minute acclimation period, we opened the isolation chamber so that emergence was possible. We gave the fish 20 minutes to emerge, as fish that do not emerge within 20 minutes are unlikely to emerge even after 60 minutes[2]. Throughout the trial, fish were recorded

from 56 cm above using a web camera (C922x Pro Stream, Logitech, Suzhou, China) at 1280 x 720 pixel resolution and 30 frames per second. After the trial concluded, fish body size was measured on graph paper to the next millimeter (standard length: from the tip of the snout to the base of the tail fin) and weighed to the next milligram on a digital scale (M-Power AZ153, Sartorius, Göttingen, Germany). Experimental tanks were always throroughly cleaned to remove residual cues. Given that we know that in the absence of risk, body size is correlated with the speed of exploration (referred to as boldness in[1]) and we therefore aimed to specifically test this relationship in the present study, we again needed to control for age effects (as age is correlated with body size) while aiming for a reasonably large sample size. Therefore, we tested every fish only once so as to reveal individual response patterns as represented by a single measure of emergence behaviour as has been established across previous studies on the same relationship[1,3,4]. As we did not test individuals repeatedly, we cannot draw any conclusions as to how the risk treatment combinations here may impact personality[5]. Additionally, as we tested sexually immature individuals where no sexual dimorphism was observable, we cannot exclude the possibility that patterns of exploratory behavior may change following sexual maturation[6]. In total, we tested 1100 minnows from 69 different clutches, however in some instances fish emerged during the acclimation time by forcing themselves out of the isolation chamber, in other cases video files were not playable due to technical issues and sometimes the experiment was disturbed by people entering the experimental room. Thus, we had to exclude 100 trials from our final

sample and analyzed data from only 1000 fish (65-142 individuals per treatment, see Fig. 4 of the main manuscript).

References

1 Meuthen, D., Ferrari, M. C. O., Lane, T. & Chivers, D. P. Plasticity of boldness: high perceived risk eliminates a relationship between boldness and body size in fathead minnows. *Anim. Behav.* 147, 25-32 (2019).

2 Poulin, N. P. N. *The effects of boldness on threat-sensitive decisions in fishes*, MSc thesis, University of Saskatchewan (2016).

3 Brown, C. & Braithwaite, V. A. Size matters: a test of boldness in eight populations of the poeciliid *Brachyraphis episcopi*. *Anim. Behav.* 68, 1325-1329 (2004).

4 Brown, C., Jones, F. & Braithwaite, V. In situ examination of boldness-shyness traits in the tropical poeciliid, *Brachyraphis episcopi*. *Anim. Behav.* 70, 1003-1009 (2005).

5 Heynen, M., Borcherding, J., Bunnefeld, N. & Magnhagen, C. Plasticity and consistency of behavioural responses to predation risk in laboratory environments. *J. Zool.* 300, 228-235 (2016).

6 Cabrera, D., Nilsson, J. R. & Griffen, B. D. The development of animal personality across ontogeny: a cross-species review. *Anim. Behav.* 173, 137-144 (2021).
